# Recent Advances in Molecular Tools and Pre-Breeding Activities in White Lupin (*Lupinus albus*)

**DOI:** 10.3390/plants14060914

**Published:** 2025-03-14

**Authors:** Andrea Tosoroni, Valerio Di Vittori, Laura Nanni, Evan Musari, Simone Papalini, Elena Bitocchi, Elisa Bellucci, Alice Pieri, Sofia Ghitarrini, Karolina Susek, Roberto Papa

**Affiliations:** 1 Department of Agricultural, Food and Environmental Sciences, Marche Polytechnic University, 60131 Ancona, Italy; a.tosoroni@univpm.it (A.T.); l.nanni@univpm.it (L.N.); e.musari@pm.univpm.it (E.M.); s.papalini@pm.univpm.it (S.P.); e.bitocchi@univpm.it (E.B.); e.bellucci@univpm.it (E.B.); a.pieri@univpm.it (A.P.); 2 Società Produttori Sementi S.p.A., Via Macero n.1, 40050 Argelato, Italy; sofia.ghitarrini@psbsementi.it; 3 Legume Genomics Team, Institute of Plant Genetics, Polish Academy of Sciences, Strzeszynska 34, 60-479 Poznan, Poland

**Keywords:** legumes, plant genetic resources, landrace, lupin, plant adaptation

## Abstract

The higher adaptation of landraces to local agroclimatic conditions resulting from natural and moderate artificial selection by farmers within specific environments makes them a crucial source of alleles and genotypes for cultivation and breeding programs. Unlike modern cultivars, which have been developed under more intense artificial selective pressures, landraces exhibit a broader genetic base that has been documented in landrace collections for many crops. This review provides an overview of the importance of genetic resource valorisation in legume species, focusing on cultivated species of the *Lupinus* genus, particularly white lupin (*Lupinus albus*). On the one hand, legumes, including Lupins, are considered a crucial alternative source of protein within the framework of more sustainable agriculture. On the other hand, they are often neglected species in terms of breeding efforts, despite receiving increasing attention in recent years. Here, we also report on the latest advances in the development of genomic tools, such as the novel pangenome of white lupin and the identification of markers and loci for target adaptation traits, such as tolerance to alkaline soils, which can effectively support the breeding of *Lupinus albus*, especially for the introgression of desirable alleles from locally adapted varieties.

## 1. Introduction

Agriculture is currently facing challenges, such as climate change and the need for resilient crops, alongside the increasing demand for food, feed, and fuel, driven by a growing and increasingly wealthy population [1,2]. There is a pressing need to ensure higher quality sustainable production, including novel sources of protein and healthier food. Achieving future food security is crucial [3], particularly under the strain of planetary boundaries, while also ensuring the resilience of Earth’s systems [4,5]. In this context, to foster more sustainable agricultural practices and produce healthier food, it is essential to preserve the diversity of crop species (i.e., plant genetic resources) and to identify strategies for their maintenance, characterisation, and valorisation, with particular regard to underutilised crop species with great potential, such as food legumes. As global demand for alternative protein sources continues to rise [6], legumes not only meet this need but also offer environmental benefits such as fixing atmospheric nitrogen (N^2^), reducing the need for chemical fertilizers, enhancing soil fertility, and provides beneficial crop rotation effects, even as the market for vegetable protein expands [7]. Nonetheless, breeding efforts in food legumes remain limited, leading to their underutilisation by consumers due to insufficient improvements in quality-related traits, which in turn results in lower cultivation rates by farmers [8]. The potential of genetic resources in legume breeding remains underexploited, as their utilisation does not match their availability, leaving much of their potential untapped. However, significant efforts are underway to develop new approaches for the conservation, management, and characterisation of legume genetic resources [9]. It is important to recognise that the unexplored genetic potential of legumes could yield significant returns on research investments, likely surpassing those of other crop species whose genetic potential has already been extensively investigated [10,11]. The concept of plant genetic resources for food and agriculture encompasses genetic material of plant origin that holds current or potential significance for agricultural practices and food production [12]. This category includes landraces or farmers’ varieties cultivated on farms, improved cultivars developed through breeding programs, accessions stored ex situ in gene banks, and wild species related to crops (known as crop wild relatives), as well as wild plants harvested for food [13,14]. In other words, genetic diversity refers to the amount of genetic variability present among individuals of a variety or within a population of a species [15].

Plant genetic resources (PGRs) represent a crucial component of the planet’s extensive biodiversity and serve as the foundation for food security, nutrition, and livelihoods while also supporting the bioeconomy [16]. For a given species, these resources include domesticated materials such as cultivars, breeding materials, and local varieties (i.e., landraces), as well as their wild relatives and any source of genetic diversity, such as genetic stocks, nucleic acids, and genes [17]. The wide array of traits present in genetic resources owing to their extensive variety of alleles and genotypes, along with their capacity for adaptation, is vital for strengthening the resilience of agroecosystems, including forests, and for driving progress toward innovative and efficient agro-food systems and bio-based value chains. Thus, the maintenance of PGRs serves as an essential component for developing new crop varieties that promote sustainable agriculture in the face of climate change and support healthy diets for a rapidly growing global population [18]. The term variety refers to a plant group distinguished by specific heritable traits that remain stable through propagation. Cultivated varieties are generally divided into two categories: “modern officially released varieties” and “farmers’ varieties” [19]. Modern varieties, developed by professional breeders in private companies or public research institutions, often exhibit a high degree of genetic uniformity and produce offspring that consistently share the same phenotypic traits, with genetic diversity that is present only between different varieties. In contrast, farmers’ varieties, or landraces, result from generations of farmers’ deliberate or unintentional selection and local adaptation, leading to significant genetic diversity both within and between varieties [20]. Landraces have emerged through a combination of natural and artificial selection by farmers in specific environments and are thus highly adapted to local conditions [21]. However, because landraces have been developed under lower selection pressure compared to modern cultivars, they collectively display a broader genetic base [22]. Crop wild relatives also serve as a valuable source of heritable traits, including those that enhance tolerance to environmental stresses and improve nutritional quality. These traits have been successfully integrated into elite crop varieties [23]; indeed, many studies have examined landrace collections and identified novel alleles for tolerance to abiotic and biotic stress, which could be used by breeding programs to improve crops [24].

It is also important to note that breeding efforts are usually conducted under controlled growing conditions (e.g., pesticide, fertilization), reducing the potential utilisation of specific landraces that could instead be particularly useful in marginal or low-input agricultural systems [25].

Regarding the collection of genetic resources [26], FAO statistics [12] indicate that more than 2500 botanical gardens and 1750 gene banks exist worldwide [27].

To support a global system for the conservation and use of crop diversity [28], the Global Crop Diversity Trust (Crop Trust) was established in October 2004 by the Food and Agriculture Organization of the United Nations (FAO) and Biodiversity International on behalf of CGIAR [29,30]. The Crop Trust is a non-profit international organisation dedicated to the conservation of crop diversity and to make it available for global use forever and for the benefit of all. For instance, the 11 CGIAR gene banks conserve 736,210 accessions of cereals, grain legumes, forages, tree species, root and tuber crops, and bananas. Many of these accessions are crop wild relatives [31]. This is the largest and most widely distributed collection of genetic diversity available under the Multilateral System of the International Treaty on Plant Genetic Resources for Food and Agriculture (ITPGRFA). Over the past 10 years, CGIAR gene banks have distributed more than an estimated million samples to plant breeders and crop researchers. From 2012 to 2020, more than 897,338 samples have been distributed to 160 countries [32].

Two main conservation approaches are recognised for crop diversity: in situ and ex situ [33,34,35,36]. The International Treaty defines the ex situ conservation as “the conservation of plant genetic resources for food and agriculture outside their natural habitats”, while the in situ conservation is defined as “the conservation of ecosystems and natural habitats and the maintenance and recovery of viable populations of species in their natural surroundings and, in the case of domesticated or cultivated plant species, in the surroundings where they have developed their distinctive properties” [37].

Ex situ conservation is usually carried out at gene banks and botanical gardens. Different types of gene banks have been established to store plant diversity, depending on the type of plant material that is conserved. These include seed banks (for seeds), field gene banks (for alive plants), in vitro gene banks (for plant tissues and cells), pollen and deoxyribonucleic acid (DNA) banks [38]. The strategy to be adopted for the ex situ conservation is largely determined by the reproduction method of the species that is undergoing conservation, and it is based on the purpose of the conservation and the use of the plant material. Currently, about 7.4 million PGRFA accessions are conserved in over 1,750 gene banks. More than 2500 botanical gardens grow over 80,000 plant species. For example, the RBG Kew Millennium Seed Bank (MSB) of the Royal Botanic Gardens, Kew (RBG Kew) and the Svalbard Global Seed Vault (SGSV) are major global facilities in modern ex situ conservation [39]. The aim of the in situ conservation of wild species is to ensure that populations of targeted species are maintained in the natural habitats where they evolved and that their survival is not threatened. The most common strategy for in situ conservation relies on declaring natural habitats as protected sites and taking appropriate measures to ensure their conservation [37].

Landraces have been defined extensively [40,41]; as “a dynamic population(s) of a cultivated plant that has historical origin, distinct identity and lacks formal crop improvement, as well as often being genetically diverse, locally adapted and associated with traditional farming systems” [42,43] or as “a cultivated, genetically heterogeneous variety that has evolved in a certain ecogeographical area and is therefore adapted to the edaphic and climatic conditions and its traditional management and uses” [44].

Thus, landraces or old cultivars can represent a valuable source of genetic variation [45,46,47]. However, such material is rarely used in breeding programs, which typically target elite × elite crosses, to improve the likelihood of developing higher yielding cultivars [48]. Here, we discuss the relevance of identifying strategies for PGRs valorisation, focusing on legume species, particularly lupins. White lupin (*L. albus*) is a promising high-protein crop that thrives in acidic soils but can also tolerate mildly alkaline and slightly calcareous conditions, with a preferred soil pH of 6.5 or lower [49]. Although some cultivars of white lupin exhibit greater tolerance to salinity and heavy soils than other crops, overall, the species’ distribution remains significantly limited by its specific growth requirements and lack of adaptation to alkaline and calcareous soils [49,50].

## 2. PGRs in Food Legumes and Plant Breeding Perspective

Food legumes play a crucial role in addressing various agricultural-related societal challenges, including climate change mitigation, agrobiodiversity conservation, sustainable agriculture, food security, and human health. The transition to plant-based diets, primarily based on food legumes, represents a significant opportunity for adaptation and mitigation while generating co-benefits for human health. By enhancing the cultivation and utilisation of legumes, we can promote healthier diets while contributing to climate change mitigation efforts [9]. This transition is particularly relevant and urgent, however, investment in breeding research for food legumes has been relatively low, resulting in significant untapped genetic potential for these crops. Currently, the European Union allocates only 3% of its arable land to protein crops and relies on imports for more than 75% of its plant protein needs. This includes approximately 400,000 tons of common beans, 200,000 tons of lentils, and 150,000 tons of chickpeas, annually [51]. The EU’s low self-sufficiency in plant protein is attributed to the delayed development and inadequate adaptation of protein crops in Europe (COPA-COGECA report, GOL (18)585), alongside the insufficient breeding efforts to adapt legumes to European agro-ecosystems. The exploitation of PGRs in food legume breeding is limited compared to the available genetic materials, resulting in suboptimal utilisation of their potential benefits.

In recent years, numerous research projects have been initiated to study pulses and address these gaps. Global market trends and increasing consumer demand for alternative plant proteins, food and sustainable diets [52,53,54] have further driven these research efforts. Many of these projects focus on enhancing legumes cultivation, including lupin species, to improve food security through increased pulse production and promote sustainable agricultural practices across various regions. Below, we summarise the relevant goals of selected projects that focused on characterisation and utilisation of the lupin genetic resources in plant (pre) breeding programs:

1. Legume Generation (2023–2028) aims to boost the breeding of major food and feed legume crops in Europe to address the protein gap and support biodiversity, such as soybean, lupin, pea, lentil (blue, white and Andean), common bean and clover. The project involves 32 partners from 16 countries. It is funded by the European Union (EU) and UK Research and Innovation, with a total budget of EUR 8.6 million [55].
2. BELIS (2023-2028) focuses on enhancing the competitiveness of the legume breeding industry in the EU and associated countries. The project aims to develop cost-effective breeding methodologies, improve regulatory frameworks for variety registration, and facilitate innovation transfer among breeders, seed industries, and agricultural stakeholders. By promoting sustainable practices and increasing the availability of high-quality legume varieties (14 species), BELIS seeks to support the production of food and feed while delivering ecosystem services, working both on forage crops (lucerne, red clover, white clover, annual clovers, sainfoin, birdsfoot trefoil and vetches) and grain crops (pea, fava bean, soybean, white lupin, lentil, chickpea and common bean) [56].
3. EUROLEGUME (2014–2017) focused on enhancing legume cultivation in Europe through sustainable cropping practices, selection, characterisation and the identification of genetic resources for fava beans, peas, and cowpeas, with the goal of improving their production, developing novel food products and optimising agricultural management for different agro-climatic conditions [57].
4. ABSTRESS (2012–2016) aimed to improve legume crop resistance to combined abiotic and biotic stresses, delivering new plant varieties with greater drought and disease tolerance. This initiative involved multiple national and international partners and sought to enhance the sustainability of European agriculture by reducing reliance on imported protein sources. The project primarily focused on legume species, including pea (*Pisum sativum*), and *Medicago truncatula*, a small low-growing clover-like legume native to the Mediterranean region. The last is a fast-growing plant commonly used as a model for legume crops [58].
5. EXTRULEG (2013–2017) developed extruded products from legumes (i.e., pea, common bean, carob) and cereals (i.e., rice), through fortification using dietary fiber, and it focused on the nutritional impacts and childhood obesity prevention. The project explored innovative food processing methods to enhance the nutritional value of legume-based snacks [59].
6. CropExplore (2018–2022) explored alternative raw materials for the food sector, focusing on nutritional qualities and functionality of various legumes. The project sought to create a knowledge matrix of known and lesser-known crops/raw materials [59].
7. LupinChallenge (2012–2015) characterised beta-conglutin seed proteins in lupin, to assess their nutritional value and impact on human health, including allergenicity. Overall, this project sought to improve lupin as a safe and nutritious plant-based protein source [60].
8. LegumES project (2024–2027) develops multi-actor driven approaches for monitoring the benefits of legume crops, focusing on beans, chickpea and lentil), legume-based crop rotations and wild legumes in semi-natural ecosystems [61].
9. EVA Legumes Network (2024–2027) aims to increase the use of crop genetic diversity in legume breeding and promote legume diversity for sustainable plant-based protein production in Europe [62]. The project covers seven legume crops: beans, chickpeas, fava beans, lentils, lupins, peas, and orphan legumes.
10. LegValue project (2017–2021) focused on enhancing the economic viability and sustainability of legume production, addressing the protein gap in European agriculture by improving the quality and marketability of several legume species (i.e., fava beans, lucerne, lupins, peas and soybeans) and promoting their cultivation and consumption. The project involved collaboration among researchers, farmers, and industry stakeholders to develop innovative practices and products [63].
11. Legumes Translated (2018–2022) supported the production and use of grain legumes, helping farmers benefit from relevant research, particularly those funded by the European Union [64].
12. LEGUMINOSE (2022–2026) promotes legume-based intercropping as a climate-smart farming practice, providing science-based and economically viable systems for European farmers. The project relies on different species and mixture according to the country where the research is conducted (e.g., in Poland, a common intercropping mixture involves cereals, such as oats, barley, wheat, and sometimes maize, paired with legumes such as peas, lupins, fava beans, and sometimes soybeans). The project will (1) investigate the benefits of intercropping beyond the well-studied effects on nitrogen dynamics, (2) identify obstacles and issues related to the adoption of the intercropping practice, and (3) provide farmers across the EU with accessible, actionable and science-based information for a profitable and sustainable agricultural transformation [65].
13. DemoNetErBo (2016–2021) established a knowledge transfer network for field peas and fava bean cultivation and utilisation, connecting conventional and organic farmers. The project aimed to build value chains, from breeding to consumer usage, meeting the increasing demand for non-GMO protein crops [66].
14. ProVeg (2017-still ongoing) is a global initiative promoting plant-based and cultivated foods, reducing global animal product consumption by 50%, by 2040. ProVeg engages with various stakeholders to foster awareness and facilitate the transition to sustainable diets, emphasising the benefits of healthy plant-based alternatives [6].
15. BRESOV (2018–2022) enhanced organic vegetable production by improving breeding and availability of organic seeds for broccoli, snap beans, and tomatoes. The project aimed to explore genetic diversity and increase the crops’ resilience to biotic and abiotic stresses, adapting them to organic farming practices. By collaborating with researchers, breeders, farmers, and stakeholders across Europe, Africa, and Asia, BRESOV promoted sustainable agriculture to meet the growing demand for high-quality organic vegetables [67].
16. BEAN ADAPT (2015–2018) contributed to improving resilience and adaptability of common bean (*Phaseolus vulgaris*) varieties to climate change and variable growing conditions. The project led to a publication in which the complex evolutionary history of common bean has been further dissected, shedding light on the mechanisms behind the successful introduction, dissemination and adaptation of common bean in Europe [68]. Collaborating with farmers, researchers, and industry stakeholders, BEAN ADAPT seeks to improve food security and sustainability in bean production systems across different regions [69].

Among the projects focused on PGRs in legume species, the H2020 INCREASE project—Intelligent Collections of Food Legumes Genetic Resources for the European Agrofood System—[9] was established, following the BEAN ADAPT project. INCREASE focuses on the conservation, management, and characterisation of PGRs for chickpea (*Cicer arietinum*), common bean (*Phaseolus vulgaris*), lentil (*Lens culinaris*), and lupins (*L. albus* and *L. mutabilis*). Its objective is to promote agro-biodiversity utilisation in Europe and beyond, as well as the sustainable use of these PGRs through efficient conservation tools and methods that could be applied to all kinds of genetic resources. The project aims to generate extensive genotypic and phenotypic data, developing *Intelligent Collections* (ICs) of inbred (i.e., pure lines) lines, and enhancing conservation through participatory citizen science approaches. A web-based tool will facilitate access to new knowledge, such as gene discovery, while promoting best practices for genetic resource management across global institutions. The project also explores decentralised data-sharing methods for more efficient conservation.

The INCREASE project’s Intelligent Collections (ICs) are structured as nested core collections of genetically purified crop accessions [70], which “memorise” genotypic and phenotypic data, “learn” from data analysis, and “evolve” through refined sampling. ICs are divided into three categories: R-CORE (large set of purified accessions), T-CORE (smaller set for detailed phenotypic and genomic analysis), and H-CORE (small set for deep analysis). Extensive phenotyping efforts include multi-location field trials, metabolomics, transcriptomics [71], and studies of root traits and diseases. Regarding phenotyping, detailed protocols for plant traits have been developed during INCREASE for the four target species at the seed multiplication stage [70,72,73,74]. The project has also adopted participatory approaches with the aim of engaging stakeholders, such as breeders, scientists, farmers, and both agrifood and non-food industries. A Citizen Science Experiment (CSE) for testing conservation and characterisation of genetic resources has been established within INCREASE; more than 21,000 registered European citizens participated across four rounds (i.e., four years). Through the CSE experiment, Citizens provided phenotypic data and images of a large set of SSD accessions (1126) of common beans. Thus, data from an unprecedented number of environments across Europe is available for a large set of common bean genetic resources, which will support the development of crop performance prediction models.

The germplasm characterisation, management and exploitation approaches developed in these projects lay a foundation for integrating increasingly accessible genomic resources with advanced ‘-omics’ analyses. This integration will accelerate the discovery of agricultural diversity and identify valuable sources for breeding programs in food legumes and other crops.

Finally, The International Legume Society (ILS), established in 2011, continues the legacy of the former European Association for Grain Legume Research (AEP). The ILS serves as a global hub for legume research and information exchange, linking agricultural research on genetic improvement, agronomy, and utilisation of grain and forage legumes across different regions of the world [75].

## 3. Lupin: A Large Genus of Interesting and Underutilised Species

Lupin is an underutilised crop genus, despite its remarkable agronomic and nutritional features; this has sparked growing interest in its potential as a valuable protein source [76].

Incorporating lupins into the diet, alongside more familiar legumes, can enhance the diversity of plant-based nutrition.

*Lupinus* is a large genus within the legume family, consisting of approximately 270 species [77]; subsequently it has been demonstrated that it includes more than 1000 species [78], distributed around the world, with most species in America (New World lupins) and only a few in Europe and the Mediterranean basin (Old World lupins; OWLs). The genus originated in the Old World and was subsequently dispersed throughout the New World [79] across various climatic zones thanks to its adaptability [77,78].

Plants of the *Lupinus* genus (lupins; Genisteae tribe) belong to the important group of grain legumes, along with those of the genera *Glycine*, *Phaseolus*, and *Arachis* [80]. Lupins are a source of valuable protein, oils, and bioactive compounds, such as isoflavones and oligosaccharides. Additionally, lupins contribute to the productivity and quality of agricultural soils, and thus play a substantial role in crop rotation.

OWLs comprise 12 autogamous species, including three crops: *Lupinus angustifolius* (narrow-leafed lupin), *Lupinus albus* (white lupin), and *Lupinus luteus* (yellow lupin). The OWLs are geographically and phenotypically diverse, mainly represented in Mediterranean regions and North Africa, highlighting their adaptation to different environments [80]. Four species among them are worth mentioning for their quality and adaptation, making them suitable for cultivation in novel environments:

- *L. albus* (white lupin) is an annual crop (2*n* = 50) with a relatively small genome size (∼580 Mbp), mostly autogamous and its domestication started about 3000–4000 years ago in the Mediterranean region [81].

- *L. mutabilis*, also called Tarwi, Andean or pearl lupin is an annual species (2*n* = 48), with a genome size of ∼930 Mbp. It is mostly autogamous and was domesticated in the Andes between 1800 and 2600 BC in the highlands of northern Peru [82].

- *L. angustifolius*, also known as narrow-leafed lupin, is an annual species (2*n* = 40) with a genome size of ∼960 Mbp. It is mainly autogamous and was probably domesticated in the Mediterranean region [83].

- *L. luteus*, also called yellow lupin, is an annual species (2*n* = 52), with a genome size of ∼962.97 Mbp. It is mostly autogamous and was probably domesticated in the Mediterranean region [84].

Further research may be necessary, as the search results did not provide specific domestication dates for these last two species.

Lupin seeds are some of the most appreciated legume seeds since they are an outstanding source of nutrients, mainly proteins, lipids, dietary fibre, minerals and vitamins [85,86]. After the alkaloids are removed from seeds by debittering or through breeding programs to obtain sweet varieties, lupin seed becomes highly valuable as human food and animal feed, due to low levels of antinutritional factors including phytates, protease inhibitors and lectins. The nutritional values for lupin species of interest and other relevant legumes are provided in Table 1 [87,88,89,90,91,92].

In particular, the protein content of white lupin is approximately 38–44% on a dry weight basis (dw), which is similar to soybean (12.5 g/100 g) and higher than other legumes, such as lentils (9.1 g/100 g), pea (6.2 g/100 g), chickpea (8.4 g/100 g) and fava bean (6.7 g/100 g) [91].

Along with macronutrients, lupin seeds are abundant in starch, fibre, antioxidants, minerals, and vitamins. Lupin seeds display a high proportion of unsaturated fatty acids, low erucic acid, and a long shelf life [77,93]. Their consumption has been demonstrated to be beneficial in lowering blood cholesterol, contributing positively to cardiovascular disease protection and antidiabetic activity [94,95,96].

**Figure 1:**
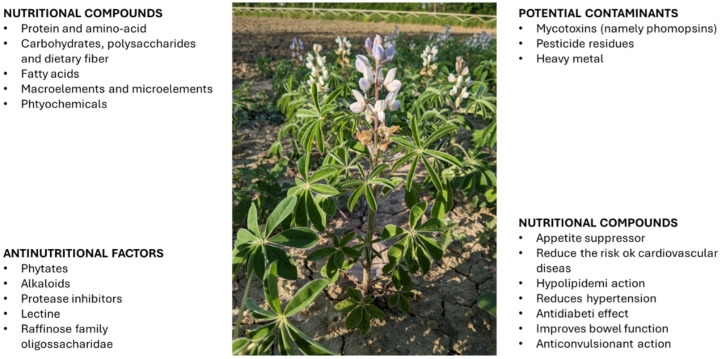
Summary of the main components of *L. albus* (from [91] with modifications).

It is worth mentioning the presence of antinutritional components, such as alkaloids [97] whose threshold for human consumption is 0.02% (DM weight) in seeds [98]. This suggests the need to identify accessions with low alkaloid content and/or ensure proper removal of such compounds before consumption, which might limit their utilisation. A summary of the main components found in white lupin seeds is provided in Figure 1.

In terms of lupin’s agronomic value, it is worth mentioning its atmospheric nitrogen fixation capability [99], significant biomass production, and ability to mobilise soil nutrients effectively [100]. This includes the interaction between nitrogen and phosphorus in the metabolism of nodule organs (N) and cluster roots (P); phosphorus (P) is required for plant growth and is often present in the soil in unavailable forms, such as phytic acid, or calcium (Ca), iron (Fe), and aluminium (Al) phosphate [100], which are difficult for plants to absorb. About 5.7 billion hectares of land worldwide contain too little P for crop growth, constraining agricultural productivity. To cope with P-limiting environments, plants have evolved several adaptations, including morphological, physiological, biochemical, and molecular mechanisms [100]. An important adaptation to these P-poor environments is the development of a functional root system with cluster roots. Essentially, a cluster root is a lateral root along which hundreds of very short rootlets develop, forming one to several very dense clusters that drastically improve soil exploration and nutrient acquisition (mostly phosphate), acidifying the surrounding area to dissolve soil P and make it available for plant absorption [101,102,103,104]. This is a crucial feature in modern agriculture, where input reduction is needed. This legume crop has high phosphorus (P)-use efficiency (PUE) in low-P soils and serves as a model crop for studying plant adaptation to low-P availability [100]. The ability of *L. albus* (and other Lupin species) to create cluster roots as a response to Al toxicity has also been demonstrated [105]. These modified roots exude different secretions, including phenolic compounds and organic acids, which detoxify Al by chelating it into non-phytotoxic complexes in the rhizosphere and vacuoles of root cells. Moreover, symbiotic rhizobia assists plants with Al detoxification.

The remarkable ability of white lupin to develop cluster roots has been observed in plants from ten different botanical families, including monocots like those in the Cyperaceae family. This raises the question of whether these structures evolved independently multiple times due to the lack of mycorrhizal associations in these species or originated from a common ancestor and were later lost in most plants. While limited genomic data for many lupin accessions hinders their use and further research, the high-quality genome sequence of white lupin—an annual crop that produces cluster roots and requires less phosphate fertiliser—will offer valuable insights into the molecular mechanisms behind these unique adaptations [106]. Given that phosphate is a limited resource, enhancing phosphate acquisition could be a significant trait for improving nutrient uptake in other crops [107]. In [107], the authors demonstrated that the breeding accessions establish the root system earlier through lateral and cluster root formation, which was indirectly selected. A closer look at these chromosome regions with low nucleotide diversity and high genetic differentiation could support the discovery of new genes with important roles in the root architecture of white lupin [107]. Hence, integrating the information from studies of gene function and the high density of variants described in the pangenome work can provide a complementary approach to forward genetic studies and contribute to the development of the research and breeding of white lupin [106]. Additionally, lupins are well-suited to rainfed agricultural systems [108] and are tolerant to drought [109]; however, so far, breeding efforts have rarely been intensive or sustained over long periods. As a result, lupins, and white lupin yields in particular, remain low and highly variable compared to other pulses like soybean for which intensive and sustained breeding efforts have been made. Although white lupin cultivation represents a promising crop for Europe, in a political context aiming towards plant protein independence from American soybean imports, the lack of well-characterised genetic resources has hampered the rapid deployment of white lupin as an alternative crop to soybean imports. Europe’s reliance on soybean imports for plant protein—covering 70% of its needs—has raised concerns about trade agreements and quality standards that do not meet citizens’ expectations. Native European legumes like white, yellow, and narrow-leafed lupins offer a promising alternative, boasting high protein content (up to 44%), health benefits, and sustainability. However, lupin cultivation in Europe is insufficient to meet the food industry’s demands, necessitating innovation in producing appealing, protein-rich lupin-based foods.

As highlighted by different works [110], a comprehensive approach is needed to establish lupins as a viable protein source, including advanced breeding techniques to develop new varieties, optimising processes for high-quality lupin protein and creating marketable products for consumers. With these strategies, lupins could enhance both socio-economic growth and environmental sustainability in Europe. As the global population’s protein demand grows, the reliance on animal products is becoming unsustainable. While lupins have thrived in Australia, where a significant industry has emerged, European production is limited and inconsistent, despite high potential yields in certain areas [111]. Overall, total lupin production reached 1,644,691 tonnes in 2022, covering an area of around 948,631 hectares worldwide, with Australia leading as the largest producer at 58.2%, followed by Europe at 34.7% [112]. This is 13.8% more than produced in the previous year and 51.2% more than 10 years ago. Additionally, it is interesting to highlight that in Europe, around 2 million tons of lupin seeds are produced annually, with 500,000 tons of lupin-based products consumed. These products include lupin bran, flour, milk, and tofu, used in various foods like biscuits, bread, sauces, and snacks [113]. From 1994 to 2020, Europe contributed 18% of global lupin production. The global lupin market is expected to grow significantly, with a projected expansion of 4.45% from 2022 to 2029 [114].

## 4. White Lupin

As stated above, white lupin (*L. albus* L.) is a pulse crop that has been domesticated starting around 3000–4000 years ago in the Mediterranean basin [81] and has always been cultivated for its seeds, which contain high levels of proteins and are used both for food and feed [115]. Wild forms of white lupin (var. *graecus*) can only be found in Greece and the adjoining Balkan region, with the earliest evidence of its use as a green manure and grain crop from that same region [78]. Early Greek farmers selected larger seeds and white flowers, and presumably, removing seed dormancy (water-permeable seeds) was the earliest domestication trait. Greek and Roman literature suggests that seed indehiscence (i.e., resistance to pod shattering) had not yet been incorporated by the first century A.D. [77,116].

The recent sequencing of the white lupin genome [100,107] and assembly of the Pangenome [106] demonstrates a resurgence of interest in this ‘old’ crop; white lupin intra-genomic diversity might reflect the early traces of its slow and sporadic domestication history [106].

**Figure 2:**
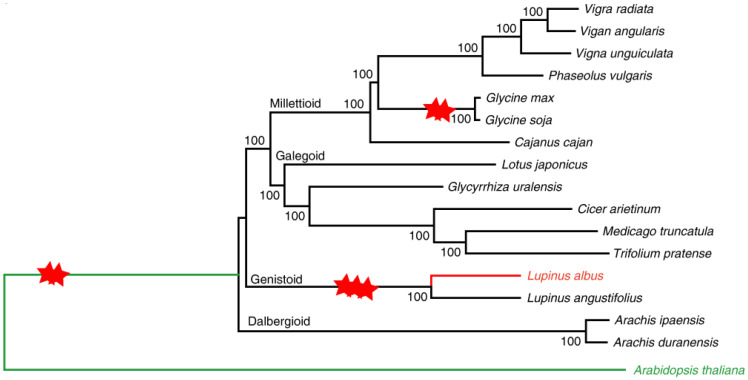
Phylogenetic tree of 16 Legume species. The two red stars represent the whole-genome duplication event, while the three red stars represent the whole-genome triplication event. *Arabidopsis thaliana* represents an outgroup species in the analysis and was highlighted using a green font, while *L. albus* has been highlighted using a red font (from [100]).

In a 2020 study [100], the genome of white lupin was assembled, revealing that it has evolved from a whole-genome triplication (WGT). *Lupinus albus* and *L. angustifolius* may have experienced a common WGT event (Figure 2): phylogenetic tree of 16 legume species built on synonymous sites from syntenic homologous genes [100].

Interestingly, they detected a new whole-genome duplication (WGD) in legumes: so far, only two events have been known for soybean [117].

**Figure 3:**
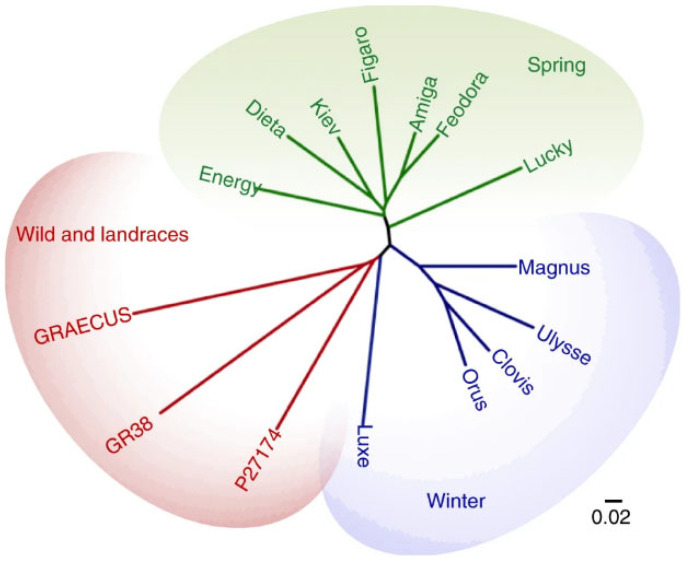
Neighbor-joining phylogenetic tree of white lupin accessions based on SNPs (from [107]).

In the work of Susek and collaborators [118], the analysis of lupin gene families provided insights into their relationship with phenotypic diversification and species adaptation, which will facilitate the exploitation of underutilised legume species by identifying genes for use in crop breeding programs. The expansion and contraction of gene families involved in seed size, a paradigmatic domestication trait, indicate that gene duplication may have led to morphological adaptations in *L. cosentinii* and *L. digitatus* differing from those in *L. albus*. However, additional work is needed to validate these hypotheses. In the work of Hufnagel and collaborators [107], a pairwise dissimilarities analysis among genotypes of *L. albus*, classified based on their biological status, has been conducted, which allowed the identification of three clusters (Figure 3), and reflect the recent breeding history of white lupin: winter accessions (responsive to vernalisation, slow-growing, and cold-adapted), spring accessions (unresponsive to vernalisation, fast-growing, vigorous, and with a shortened life cycle), and landraces/wild types.

In a more recent study [106], the *L. albus* pangenome has been assembled, highlighting the species’ diversity, including single nucleotide polymorphisms (SNPs) and gene presence–absence variations (PAVs). The pangenome comprises ‘core’ genes found in all individuals and ‘variable’ genes that may be absent in some. Utilising this extensive dataset, they identified selection markers for low seed alkaloid content and candidate genes linked to these traits. Their analyses offer fresh insights into the intra-species diversity and domestication history of white lupin. Hufnagel et al. (2021) [106] gathered 39 white lupin accessions, including 25 modern cultivars, 10 landraces, and four wild accessions from 17 countries, to represent the species’ diversity. Genome sequences for 15 accessions were previously available [107], while 24 were sequenced in this study. The de novo assembly produced 14.9 Gb of contigs longer than 500 bp, with an N50 of 24,475 bp and a mean BUSCO completeness score of 96.3%. The pangenome was constructed using a ‘map-to-pan’ approach, revealing 270 Mb of non-reference sequence. After refining the assembly, they generated 3663 scaffolds totalling 11,733,253 bp and identified 178 newly predicted protein-coding genes. The complete white lupin pangenome, including both reference and non-reference sequences, is 462,705,661 bp and contains 38,446 protein-coding genes, aligning with nuclear DNA estimates, which suggests that it represents the complete genome sequence of the species.

**Figure 4:**
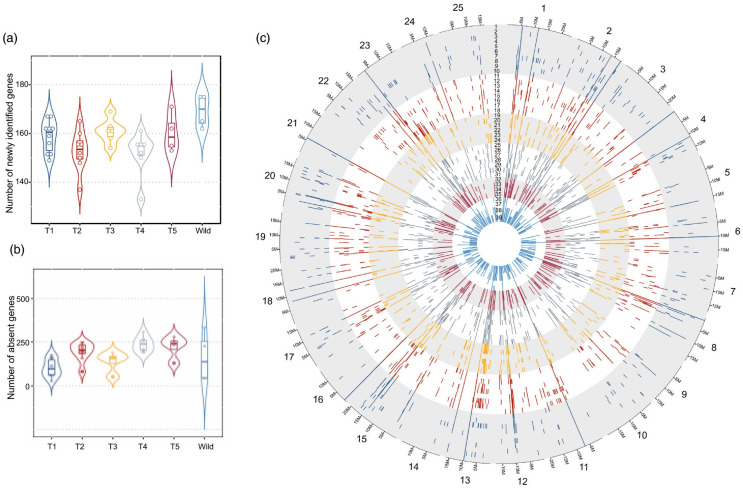
PAV of coding gene in *L. albus*. (**a**) Number of newly identified genes by phylogenetic group. (**b**) Number of absent genes by phylogenetic groups. (**c**) Positioning of absent genes in the 25 white lupin chromosomes in each 1 of the 39 accessions. Order of accessions from outer to inner track: 1-AMIGA, 2-FEODORA, 3-FIGARO, 4-ENERGY, 5-KIEV MUTANT, 6-HANSA, 7-P21525, 8-PRIMORSKY, 9-DIETA, 10-VOLODIA, 11-START, 12-N3507, 13-TOMBOWSKIJ, 14-KALINA, 15-SYR6258B, 16-LUCKY, 17-MURRINGO, 18-SHINFIELD, 19-ALB01, 20-LUXE, 21-ULYSSE, 22-MAGNUS, 23-CLOVIS, 24-ORUS, 25-NAHRQUELL, 26-GYUNLATANYA, 27-NEULAND, 28-NEUTRA, 29-BADAJOZ, 30-EGY6484B, 31-POUTIGANO, 32-P27174, 33-GERELTA, 34-DOGAN, 35-WADO, 36-GR38, 37-GRAECUS, 38-BATSI, and 39-GRC5262B. The accessions’ colours reflect the six idiotypes. (from [106]).

Presence and absence variants (PAVs) are significant forms of structural variation that contribute to genomic and phenotypic diversity. The construction of a white lupin pangenome revealed 1195 PAVs that are at least absent in one of the accessions, with 1132 from the reference genome and 63 from newly identified genes (Figure 4).

Analysis indicated that wild accessions have a notably higher number of newly identified genes, with some accessions missing only a few. The number of missing genes varies between accessions, from 45 to 348. Each phylogenetic group shares a median of 31 common missing genes, and 103 genes are absent in at least one accession from each group. The wild group has 137 exclusively lost genes, while Ethiopian landraces share 118 common missing genes, primarily concentrated on chromosome 17. Chromosomal analysis showed specific patterns of PAV distribution, particularly on chromosomes 13 and 4, where certain regions are missing in multiple accessions. Chromosome 23 has the highest number of PAVs across all groups. Functional analysis of these PAVs indicated enrichment for genes associated with membrane components and oxidation-reduction processes, which are often linked to stress responses. The presence or absence of these genes may be influenced by evolutionary adaptations and a historical whole-genome triplication event in white lupin [107].

The gene loss associated with the domestication process is in line with what has been observed in the recent assembly of the common bean pangenome [119].

**Figure 5:**
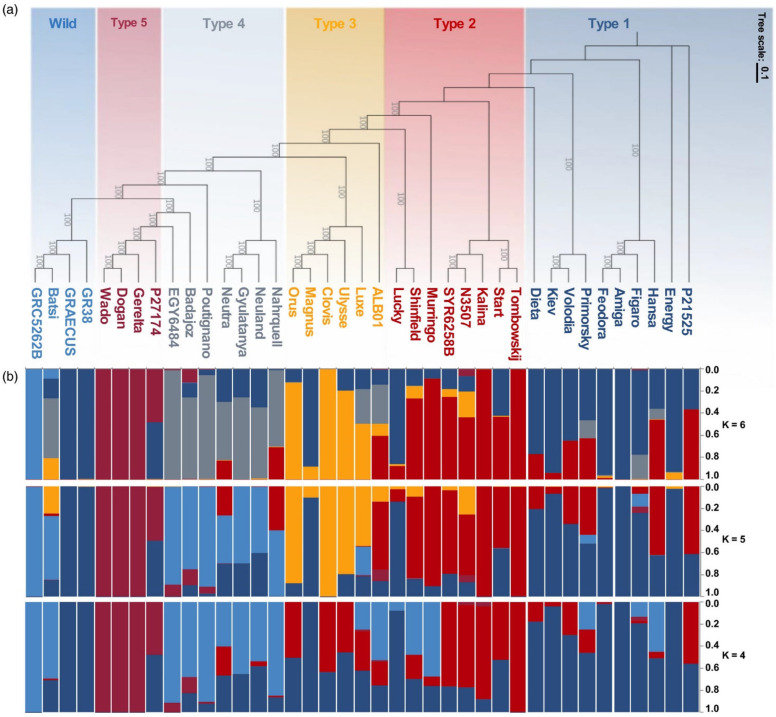
Phylogeny and population structure of 39 accessions of *L. albus*. (**a**) Maximum likelihood phylogenetic tree of white lupin constructed based on 3.5 m SNPs. The accessions are divided into six idiotypes, represented with different colors. (**b**) Model-based clustering analysis with different numbers of ancestral kinships (k = 4, 5, and 6). The y-axis quantifies cluster membership and the x-axis lists the different accessions. The positions of these accessions on the x-axis are consistent with those in the phylogenetic tree (from [106]).

Analysing the genetic structure by performing a Bayesian model-based clustering analysis, Hufnagel and collaborators [106] found that the six population groups matched the maximum-likelihood tree (Figure 5a). This presented evidence of significant admixture in some lines and a weak population structure, a pattern already seen in other studies of *L. albus* [120]. This weak population structure is also seen through the population-differentiation statistic (*F*_ST_). The *F*_ST_ value between all six groups was 0.27; however, *F*_ST_ between Type 1 and Type 2 are as low as 0.086, and Type 4 and Wild have an *F*_ST_ of 0.092. Indeed, regarding the Bayesian model, in scenarios dividing the accessions into four or five sub-populations (Figure 5b, *K* = 4 and *K* = 5), accessions from Type 4 are merged with the Wild group. On the other hand, Type 5 showed a strong differentiation from the other groups, with *F*_ST_ values ranging from 0.34 to 0.46, with Type 4 and Type 3, respectively, which corroborates the results of previous studies [120].

The assembly of the white lupin pangenome offers extensive insights into genetic variation that remain largely untapped by researchers and breeders. Despite the existence of a vast collection of white lupin accessions in gene banks worldwide, these have not been thoroughly explored or genetically characterised. This pangenome will serve as a valuable resource to advance the study of white lupin as a model legume for future functional research and molecular breeding efforts. In fact, by examining chromosome regions with low nucleotide diversity and high genetic differentiation, they may identify key genes involved in the root architecture of white lupin. Combining gene function data with the high-density variant information from this pangenome offers a complementary strategy to forward genetic studies, advancing research and breeding efforts for white lupin [106].

For example, one of the most important research lines for the crop concerns the reduction/removal of bitter compounds.

Seeds from wild genotypes and landraces of white lupin may display a high content of quinolizidine alkaloids, resulting in a bitter taste and possible toxicity. Lysine-derived alkaloids are characteristic of the tribe *Genisteae* [121,122], a monophyletic basal tribe of the *Fabaceae* family. Traditionally, these bitter compounds were removed from white lupin seeds by soaking in water, which is still carried out across the Mediterranean and Nile regions [81]. However, this is uneconomic on a broad scale, e.g., for feed production, which motivated the identification of low-alkaloid mutants in Germany in the 1930s, aided by advances in chemistry [77]. Modern cultivars of white lupin incorporate low-alkaloid genes, hence the term ‘sweet’ lupins [123].

Lupin contains alkaloids primarily from the quinolizidine family, which includes around 100 bitter compounds. The alkaloid content varies by species: bitter cultivars have 0.5% to 6% alkaloids, while sweet varieties contain less than 0.02% [91].

In a wide panel, Kroc and collaborators [124] analysed the total content and qualitative composition of alkaloid in seeds from 367 *L. albus* accessions from the Polish Genebank (four classes of origin: wild collected material, landraces, breeding lines, and cultivars). They confirmed the expected broad variation, alongside a strong differentiation in the alkaloid content. A clear influence of domestication was also observed, with decreased alkaloid content in breeding lines and cultivars. The total alkaloid content varies from 0.02% to 12.73% of the seed dry weight. Six major alkaloids were revealed: lupanine (28.22–94.49%, mean 76.06% of the total content), 13-hydroxylupanine (0.10–32.78%, mean 8.23%), multiflorine (0.00–21.67%, mean 5.52%), albine (0.00–18.55%, mean 4.48%), angustifoline (0.24–12.14%, mean 2.07%), and 11,12-seco-12,13-didehydromultiflorine (0.00–12.28%, mean 1.74%). Due to its abundance, lupanine was found to be the most closely correlated with the total alkaloid content. These results have been confirmed in many other works, with the major alkaloids in *L. albus* seeds including lupanin, hydroxyaphylline, albine, multiflorine, sparteine, and anagraine [125,126].

These alkaloids serve as a defence mechanism against herbivores due to their toxicity and bitterness, with concentrations peaking during flowering. For humans and animals, high doses can be toxic, potentially causing symptoms like nausea, respiratory issues, and liver damage [91]. To avoid potential health risks with lupin’s alkaloids consumption, several countries have set maximum limits for alkaloids in lupine products, such as 200 mg/kg in France, Great Britain, Australia, and New Zealand. However, the European Union currently lacks specific limits for these compounds in food, although regulations require that contaminants be kept as low as possible to protect public health. At a general European level, according to the Annex of Commission Regulation (EC) No. 1881/2006, which establishes maximum levels (MLs) for the same contaminants in different foods, no MLs are currently set for quinolizidine alkaloids (QAs) in food. These limits are required by Article 2 of Council Regulation (EEC) No. 315/93 (Off. J. Eur. Comm.—1993), which states that foods containing a contaminant in amounts unacceptable to public health must not be placed on the market, that contaminant levels must be maintained as low as possible, and that, if necessary, the European Commission may set maximum levels for specific contaminants [127].

The genetic control of alkaloid production in white lupin has been extensively studied, revealing several key loci and genes involved in regulating alkaloid biosynthesis. The biosynthesis of these QAs is not yet fully clarified, but recent studies suggest that between 6 and 9 enzymes catalyse the conversion of L-lysine to (−)-sparteine. The first two pathway enzymes are already known; the rest await discovery [128].

The *pauper* locus is a major gene associated with alkaloid content in *L. albus* [110,129]. The cultivated varieties have been shown to carry a naturally occurring mutation at this locus, which decreases QAs levels below the 0.02% threshold, established as safe for consumption as food and feed. However, fundamental knowledge is required to maintain a reduction of QAs within breeding programs [130].

This locus has been crucial in breeding programs to develop “sweet” cultivars with low alkaloid levels, making them suitable for human consumption and animal feed. Several markers for selection have been developed for this region on Chromosome 18 [131,132] in correspondence with the gene cluster that co-maps with the *pauper* locus. Here, a 958-kb region contains 66 protein-coding genes [107]. Hufnagel and collaborators [106] re-sequenced 39 lines, 15 of which (including 11 modern cultivars, one landrace, and two wild accessions) had been previously adopted in the work of [107] and assembled the pangenome of white lupin. As expected, they observed a prominent peak on chromosome 18 in this region, which harbours a major QTL for low-alkaloid content, corresponding to the *pauper* locus.

Mancinotti and collaborators [123] suggested an acetyltransferase (AT) as a candidate gene for the pauper locus, where a single-nucleotide polymorphism (SNP) strongly impairs AT activity, causing a blockage in the pathway. They confirmed their hypothesis by replicating the *pauper* chemotype in narrow-leafed lupin via mutagenesis, adding a new dimension to QA biosynthesis and establishing the identity of a lupin sweet gene for the first time. This discovery facilitates lupin breeding and enables the domestication of other QA-containing legumes.

Therefore, other genes and regulatory elements could play a crucial role in achieving breeding goals, as the synthesis of QAs remains poorly understood. This knowledge gap complicates global breeding efforts for a crop like white lupin, which offers significant nutritional value. By employing advanced techniques to investigate and modify the genes responsible for alkaloid synthesis in *Lupinus albus*, we can gain deeper insights into the accumulation of secondary metabolites in lupin seeds.

Overall, the genetic landscape governing alkaloid content in *Lupinus albus* is complex but well-defined by several key loci, particularly the pauper locus, which plays a pivotal role in breeding low-alkaloid cultivars. Several studies have highlighted how alkaloid content can be affected by soil type and environmental factors [133,134] and environmental conditions [98,135]. For example, QA biosynthesis seems to be regulated by light and water conditions, with production increasing during the day and under conditions of low water availability [125].

As detailed so far, white lupin (*L. albus*) is a legume that is gaining more attention for its potential as a high-protein crop and its ability to thrive in nutrient-poor soils. However, its cultivation is often limited by specific soil adaptation requirements. The main ecological limitation to white lupin cropping likely lies in its poor adaptation to calcareous or alkaline soils [136], similarly to other relevant lupin species (*L. mutabilis*, *L. angustifolius*, *L. luteus*). Interestingly, sources of adaptation to alkaline and heavy soils have been found in the *Lupinus* genus, such as in *L. pilosus,* and *L. atlanticus* [137].

White lupin can tolerate a wide pH range, typically from 5 to 8, but faces significant challenges in alkaline soils, particularly those rich in calcium carbonate [50]. In alkaline environments, the high pH can lead to iron deficiency, resulting in chlorosis and impaired growth, as the availability of essential nutrients decreases. Although this does not seem to be the primary cause of poor growth in sensitive lupins on alkaline soils [138], it is likely associated with low rainfall in the field trial area [139], where low water content and little HCO_3_^−^; accumulation occur.

Furthermore, bicarbonates can inhibit the effectiveness of nitrogen-fixing symbiosis with *Bradyrhizobium*, which is critical for the plant’s nitrogen acquisition [49]. The interaction between soil pH, calcium levels, and nutrient availability creates a complex environment that can hinder the successful establishment and productivity of *L. albus*. Understanding these soil adaptation challenges is essential for developing effective management strategies and selecting appropriate cultivars for specific environments.

Thus, it is crucial to identify specific varieties that can withstand high calcium levels for expanding the cultivation of *L. albus* in regions with alkaline soils [140]. So far, specific varieties have been identified as better suited for these challenging environments [141].

Several studies [142,143,144] highlighted the adaptation of Egyptian germplasm to moderately calcareous soils, which reflected their adaptation to conditions of their geographic origins. Variation in adaptation to limed or alkaline soils was also reported by Liu and Tang [145] for commercial varieties and some accessions of indefinite origin, as well as within Italian landrace germplasm [50]. Another study assessed the effective utilisation of genetic diversity from landraces for yield selection and tolerance to alkaline soils [146].

The high susceptibility of Egyptian germplasm to winter cold stress limits its exploitation in breeding autumn-sown varieties for subcontinental climates or Mediterranean environments [136,140], highlighting the need for germplasm sources that combine agroclimatic adaptation to these environments with tolerance to moderately calcareous soils [147].

Both grain and biomass yields are likely to decline in soils with active lime levels exceeding 1%, which are classified as mildly calcareous, or in soils exhibiting alkaline reactions. Numerous studies, including [141], have shown that active lime exerts a more significant negative impact on crop yields than alkalinity, although these factors often coexist in calcareous soils. While calcium ions (Ca^2+^) can directly influence *Lupinus* species, the primary effects of lime are indirect. This occurs through the precipitation of organic acids released by lupin cluster roots, which are essential for mobilising and absorbing phosphorus and iron [148], and through the inhibition of iron uptake by bicarbonate ions (HCO_3_^−^) [149]. Notably, while the relative proportion of lupin cluster roots increases in response to limed soils, the overall root biomass tends to decrease [150]. Additionally, high concentrations of soil Ca^2+^ and alkaline conditions can hinder both the growth and nodulation capacity of the nitrogen-fixing microsymbiont *Bradyrhizobium* associated with lupins [151]. However, selecting lime-tolerant plant varieties may have a more significant effect on crop adaptation to calcareous soils than merely utilising *Bradyrhizobium* strains adapted to these soil conditions [147]. Phenotypic selection for white lupin lime tolerance may be complicated by high soil heterogeneity when performed under field conditions, as well as by abnormal plant root growth [152] and relatively modest correlations with genotype responses in agricultural environments when performed in pots or liquid culture [50]. Moreover, lime tolerance and adaptation to other site-specific agroclimatic characteristics are often confounded when assessing genotype yield responses under field conditions. This suggests the need to assess these responses across agroclimatically-contrasting sites that feature calcareous soil.

Despite these challenges, Annicchiarico and collaborators [50] working with a collection of 140 GBS-genotyped sweet-seed breeding lines (crossing four elite landraces × four elite sweet-seed cultivars/breeding lines), found an interesting candidate gene for improving white lupin tolerance to calcareous soils. They also suggested that lime tolerance is under polygenic control.

By assessing phenotypic variation and the trait architecture based on a genome-wide association study (GWAS), across two different growing environments, they detected a SNP on chromosome 7 that is associated with a gene encoding a putative ferric-chelate reductase (Lalb_Chr07g0184151). This enzyme has been shown to affect lime tolerance in various other species, both herbaceous [153,154] and arboreal [155,156]. In detail, the activity of this enzyme is involved in the absorption of iron, which is poorly available in soils with a high pH [157] and active lime [148], by reducing chelated Fe^3+^ according to the acquisition strategy I, that is typical of non-graminaceous species [158].

## 5. Incorporating Landrace Traits in Breeding Programs: Benefits and Limitations

The utilisation of plant genetic resources, such as landraces and wild relatives, is essential for plant breeding programs because they provide valuable genetic diversity that can be used to improve crop species [159]. As previously mentioned, these resources often contain traits not found in modern varieties, such as resistance to diseases, tolerance to environmental stress or improved nutritional quality [160]. For many species (e.g., barley, maize), it has been widely demonstrated that genetic gains can be achieved simply by using local landraces under low input and climatically marginal conditions [161]. The selection of genotypes under low input cultivation could be important to achieving success in breeding programmes that deal with strong genotype-by-environment interactions [162].

Introgression of wild genes has been successfully deployed in food legumes to develop improved varieties, pre-bred lines, genetic stocks, mapping populations, and bridge species across many species, with notable examples [163,164].

The relatively high yield of landraces and the significant genotype-by-location interaction observed in many other experiments suggest that a similar strategy could be used to develop lupin germplasm adapted to low-input growing environments. For example, this could be applied in environmentally dry conditions, such as those in Egypt [142], or more widely in the environmental conditions of Europe and Mediterranean-climate regions, relying on genetic resources, adaptation strategies and adaptive traits [108]. The outstanding agronomic value exhibited by several landrace accessions, when compared to modern cultivars, encourages their use in crosses with alkaloid-free elite cultivars or breeding lines, targeting specific agroclimatic environments. These landraces can provide a valuable genetic resource for white lupin breeding programs and the expansion of white lupin cultivation into various hardiness zones [165,166].

However, integrating these genetic resources into breeding programs comes with several challenges, including issues related to linkage drag, genetic incompatibility, and the complexity of identifying beneficial traits.

In addition, the development of pangenomes to be used as references, novel genotyping platforms, multi-omics approaches, and the available knowledge from genome-wide association studies (GWAS) can provide valuable insights into the genetic architecture of key agronomic traits. These tools help link essential genes and genomic regions to relevant phenotypes [167], thus supporting plant breeding for underexplored species, such as lupins and other legumes. Such tools and knowledge, including marker-assisted selection (MAS) and genomic selection, are crucial for tracking the introgression of both desired and undesired alleles into modern varieties and supporting breeding strategies such as pyramiding. This enhances their potential for better deployment and exploitation in breeding for more sustainable agricultural production, particularly improving adaptation and productivity in stress-prone environments to cope with climate change [168]. The problem of missing heritability in genome-wide association studies defines a major challenge in genetic analyses of complex biology. The solution to this issue is to identify all causal genetic variants and measure their contributions using a pangenome [169], thereby discovering all the genes introduced through introgression [170]. Regarding the challenges of using wild or non-elite material in breeding programs, linkage drag refers to situations where a desirable gene (e.g., associated with disease resistance) is inherited or introgressed along with unwanted genes or alleles from the donor parent, because they are genetically linked to the beneficial gene. These may not even be present in the genotype that one desires to improve. This can result in a decrease in overall performance. New innovations in breeding technology are still required to overcome linkage drag, allowing beneficial alleles to be introduced while maintaining optimal combinations [171].

Wild relatives of crops often contain traits such as pest resistance and adaptation to climate change, which can be leveraged to improve cultivated varieties. This is typically accomplished by crossing wild plants with cultivars, followed by backcrossing (introgression) to remove deleterious and unwanted genetic material from the crop. However, it can be challenging to remove all unwanted genes. A recent study [172], generated and analysed reference sequences and trait data for sunflower to examine the consequences of linkage drag. They found that crop wild-introgression had increased the genetic diversity of the crop gene pool, but had negatively affected yield and quality-related traits. In summary, by combining high-quality reference genomes, including pangenome, with genotypic and phenotypic analyses of the cultivated sunflower association mapping population, the study provides an assessment of the impact of linkage drag on the cultivated sunflower genome and on the performance of inbred sunflower lines. Despite the numerous benefits of tapping into cultivated wild relatives [173], such as the introduction of desirable traits and genetic and phenotypic variation [174], there can be downsides, including reductions in yield-related traits. The authors suggested that this is largely due to the introduction of variation in gene content; cultivars containing introgressions not only acquire new genes, but also lose genes that would otherwise be present, which can have deleterious consequences.

Marker-assisted selection (MAS) has proven effective in supporting more precise introgressions, although challenges persist, particularly in regions with low recombination and where desired and undesired alleles are tightly linked. To mitigate linkage drag, breeding efforts could focus on closely related and fully compatible wild relatives. Furthermore, natural introgression from the secondary gene pool into the primary gene pool may provide alleles that have already undergone the purging of harmful incompatibilities, thereby reducing linkage drag [172].

MAS and backcross breeding are commonly employed to reduce linkage drag. In backcrossing, the desirable gene is repeatedly crossed with the elite variety to remove unwanted genes. MAS, which uses various types of markers [175], assists breeders in identifying the desired gene without needing full phenotyping assessment, making it easier to select plants with fewer undesirable traits. Key factors for optimising MAS in backcross programs include the distance between flanking markers and the introgressed gene, population size, and the duration of the backcrossing process [176]. Additionally, a novel strategy called breeding by selective introgression (BBSI) has been proposed and implemented for the simultaneous improvement, genetic dissection, and allele mining of complex traits to realise Breeding By Design (BbD). BBSI involves three phases: (a) developing large numbers of trait-specific introgression lines (ILs) using backcross breeding in elite genetic backgrounds, which serves as the material platform for BBD; (b) efficiently identifying genes or quantitative trait loci (QTLs) and mining desirable alleles affecting various target traits from diverse donors, forming the information platform for BBD; and (c) developing superior cultivars by BBD through designed QTL pyramiding or marker-assisted recurrent selection [177]. Overall, the development of a pangenome, along with the identification of markers, QTLs, and candidate genes for target traits, as discussed earlier, are effective tools to support the development of plant breeding programs in underutilised species, such as in the *Lupinus* genus and other legumes. Breeding efforts are essential to meet the increasing demand for alternative protein sources in the context of more sustainable agriculture. Evidence suggests that introgressing desirable alleles for adaptive traits and seed quality (e.g., alkaloid content in lupins) from locally adapted cultivars or even wild relatives can be an effective strategy. However, advanced genomics tools such as MAS and available high-quality reference genomes can expedite these efforts.

Genetic incompatibility can occur when wild relatives are crossed with modern cultivars. These plants may have different genomic structures, which can lead to hybridisation difficulties, poor fertility in the offspring, or incomplete expression of desirable traits. Many wild relatives have distinct genome structures or chromosome numbers compared to domesticated varieties. This can make it challenging to obtain fertile offspring when hybridising wild relatives with modern crops. Even when hybrids are obtained, they may have low vigour or be sterile, preventing the successful transfer of beneficial genes [178].

## 6. Conclusions

In this review, we have summarised the literature on the current state of plant prebreeding and breeding in legumes, with a focus on *Lupinus* species, as well as recent developments in the conservation and the sustainable utilisation of PGRs to ensure global food security, for both direct food production and plant breeding.

While the incorporation of plant genetic resources, including landraces and wild relatives, into breeding programs is essential for enhancing genetic diversity and improving crops, it also presents several challenges. These challenges—such as linkage drag, genetic incompatibility, and the complexity of trait inheritance—can be mitigated usng advanced breeding tools.

Thus, we have outlined information on the current knowledge on tools that can effectively support plant breeding in *Lupinus albus*, including the recent development of the pangenome and the identification of markers and candidate genes for key traits, such as alkaloid content and lime soil tolerance.

## Figures and Tables

**Table 1:** Food legumes’ protein and oil content.

Species	PROTEIN %	OIL %	References
White lupin	38–44	9–13	[89,91]
Andean lupin	41–51	14–24	[89]
Narrow leaf lupin	30–35	5–10	[89]
Yellow lupin	30–40	14–24	[89]
Soybean	36.9	18.1	[89,91]
Chickpea	23.6	6.4	[87,91]
Pea	21.9	2.3	[87,91]
Lentil	20.6	2.15	[91]
Common bean	21.3	1.6	[92]
Fava bean	25	1.3	[87,91]

## Data Availability

No new data was created.

## References

[1] Grafton R.Q., Daugbjerg C., Qureshi M.E. (2015). Towards Food Security by 2050. Food Secur..

[2] FAO (2018). The Future of Food and Agriculture—Alternative Pathways to 2050.

[3] Hopf E. (2023). The Global Significance of Genetic Resources for Food Security. J. Biodivers. Bioprospect. Dev..

[4] Calicioglu O., Flammini A., Bracco S., Bellù L., Sims R. (2019). The Future Challenges of Food and Agriculture: An Integrated Analysis of Trends and Solutions. Sustainability.

[5] Gerten D., Heck V., Jägermeyr J., Bodirsky B.L., Fetzer I., Jalava M., Kummu M., Lucht W., Rockström J., Schaphoff S. (2020). Feeding Ten Billion People Is Possible within Four Terrestrial Planetary Boundaries. Nat. Sustain..

[6] ProVeg. https://proveg.org/.

[7] Green A., Blattmann C., Chen C., Mathys A. (2022). The Role of Alternative Proteins and Future Foods in Sustainable and Contextually-Adapted Flexitarian Diets. Trends Food Sci. Technol..

[8] Foyer C.H., Lam H.M., Nguyen H.T., Siddique K.H.M., Varshney R.K., Colmer T.D., Cowling W., Bramley H., Mori T.A., Hodgson J.M. (2016). Neglecting Legumes Has Compromised Human Health and Sustainable Food Production. Nat. Plants.

[9] Bellucci E., Mario Aguilar O., Alseekh S., Bett K., Brezeanu C., Cook D., De la Rosa L., Delledonne M., Dostatny D.F., Ferreira J.J. (2021). The INCREASE Project: Intelligent Collections of Food-Legume Genetic Resources for European Agrofood Systems. Plant J..

[10] Ray D.K., Ramankutty N., Mueller N.D., West P.C., Foley J.A. (2012). Recent Patterns of Crop Yield Growth and Stagnation. Nat. Commun..

[11] Semba R.D., Ramsing R., Rahman N., Kraemer K., Bloem M.W. (2021). Legumes as a Sustainable Source of Protein in Human Diets. Glob. Food Sec..

[12] FAO (2010). The Second Report on the State of the World’s Plant Genetic for Food and Agriculture.

[13] Bélanger J., Pilling D., FAO (2019). The State of the World’s Biodiversity for Food and Agriculture.

[14] Sahu P.K., Sao R., Khute I.K., Baghel S., Patel R.R.S., Thada A., Parte D., Devi Y.L., Nair S., Kumar V. (2023). Plant Genetic Resources: Conservation, Evaluation and Utilization in Plant Breeding. Advanced Crop Improvement, Volume 2: Case Studies of Economically Important Crops.

[15] Salgotra R.K., Chauhan B.S. (2023). Genetic Diversity, Conservation, and Utilization of Plant Genetic Resources. Genes.

[16] Vaccino P., Antonetti M., Balconi C., Brandolini A., Cappellozza S., Caputo A.R., Carboni A., Caruso M., Copetta A., de Dato G. (2024). Plant Genetic Resources for Food and Agriculture: The Role and Contribution of CREA (Italy) within the National Program RGV-FAO. Agronomy.

[17] Toro M.A., Caballero A. (2005). Characterization and Conservation of Genetic Diversity in Subdivided Populations. Philos. Trans. R. Soc. B Biol. Sci..

[18] Frankham R., Ballou J.D., Briscoe D.A., McInnes K.H. (2002). Introduction to Conservation Genetics.

[19] Korir N.K., Han J., Shangguan L., Wang C., Kayesh E., Zhang Y., Fang J. (2013). Plant Variety and Cultivar Identification: Advances and Prospects. Crit. Rev. Biotechnol..

[20] Berg T. (2009). Landraces and Folk Varieties: A Conceptual Reappraisal of Terminology. Euphytica.

[21] Reif J.C., Zhang A.P., Dreisigacker A.S., Warburton M.L., Van Ginkel A.M., Hoisington A.D., Bohn M., Melchinger A.A.E. (2015). Wheat Genetic Diversity Trends during Domestication and Breeding. Theor. Appl. Genet..

[22] Cavanagh C.R., Chao S., Wang S., Huang B.E., Stephen S., Kiani S., Forrest K., Saintenac C., Brown-Guedira G.L., Akhunova A. (2013). Genome-Wide Comparative Diversity Uncovers Multiple Targets of Selection for Improvement in Hexaploid Wheat Landraces and Cultivars. Proc. Natl. Acad. Sci. USA.

[23] Tirnaz S., Zandberg J., Thomas W.J.W., Marsh J., Edwards D., Batley J. (2022). Application of Crop Wild Relatives in Modern Breeding: An Overview of Resources, Experimental and Computational Methodologies. Front. Plant Sci..

[24] Kapazoglou A., Gerakari M., Lazaridi E., Kleftogianni K., Sarri E., Tani E., Bebeli P.J. (2023). Crop Wild Relatives: A Valuable Source of Tolerance to Various Abiotic Stresses. Plants.

[25] Ceccarelli S. (1994). Specific Adaptation and Breeding for Marginal Conditions. Euphytica.

[26] Plucknett D.L., Smith N.J.H., Williams J.T., Anishetty N.M. (1983). Crop Germplasm Conservation and Developing Countries. Science.

[27] FAO International Treaty on Plant Genetic Resources for Food and Agriculture. https://openknowledge.fao.org/server/api/core/bitstreams/a9d0de2a-8e98-4f75-98a8-673078841030/content.

[28] Ramanatha Rao V., Hodgkin T. (2002). Genetic Diversity and Conservation and Utilization of Plant Genetic Resources. Plant Cell Tissue Organ. Cult..

[29] FAO, CGIAR The Biodiversity Plan—Crop Trust. https://www.croptrust.org/about/the-biodiversity-plan/.

[30] Bohra A., Kilian B., Sivasankar S., Caccamo M., Mba C., McCouch S.R., Varshney R.K. (2022). Reap the Crop Wild Relatives for Breeding Future Crops. Trends Biotechnol..

[31] CGIAR Genebank Platform—CGIAR. https://www.cgiar.org/research/program-platform/genebank-platform/.

[32] CGIAR Genebank Platform. https://www.genebanks.org/genebanks/.

[33] Gepts P. (2006). Plant Genetic Resources Conservation and Utilization: The Accomplishments and Future of a Societal Insurance Policy. Crop Sci..

[34] Priyanka V., Kumar R., Dhaliwal I., Kaushik P. (2021). Germplasm Conservation: Instrumental in Agricultural Biodiversity—A Review. Sustainability.

[35] Hammer K., Gladis T., Diederichsen A. (2003). In Situ and On-Farm Management of Plant Genetic Resources. Eur. J. Agron..

[36] Rajpurohit D., Jhang T. (2015). In Situ and Ex Situ Conservation of Plant Genetic Resources and Traditional Knowledge. Plant Genetic Resources and Traditional Knowledge for Food Security.

[37] FAO Conservation and Sustainable Use Under the International Treaty. https://www.fao.org/4/i2579e/i2579e.pdf.

[38] Cohen J.I., Williams J.T., Plucknett D.L., Shands H. (1991). Ex Situ Conservation of Plant Genetic Resources: Global Development and Environmental Concerns. Science.

[39] Eastwood R.J., Cody S., Westengen O., Von Bothmer R. (2015). Conservation Roles of the Millennium Seed Bank and the Svalbard Global Seed Vault. Crop Wild Relatives and Climate Change.

[40] Zeven A.C. (1998). Landraces: A Review of Definitions and Classifications. Euphytica.

[41] Harlan J. (1992). Crops and Man.

[42] Bitocchi E., Nanni L., Rossi M., Rau D., Bellucci E., Giardini A., Buonamici A., Vendramin G.G., Papa R. (2009). Introgression from Modern Hybrid Varieties into Landrace Populations of Maize (*Zea mays* ssp. *mays* L.) in Central Italy. Mol. Ecol..

[43] Villa T.C.C., Maxted N., Scholten M., Ford-Lloyd B. (2005). Defining and Identifying Crop Landraces. Plant Genet. Resour..

[44] Casañas F., Simó J., Casals J., Prohens J. (2017). Toward an Evolved Concept of Landrace. Front. Plant Sci..

[45] Motley T.J., Zerega N., Cross H. (2006). Darwin’s Harvest: New Approaches to the Origins, Evolution, and Conservation of Crops.

[46] Jones H., Lister D.L., Bower M.A., Leigh F.J., Smith L.M., Jones M.K. (2008). Approaches and Constraints of Using Existing Landrace and Extant Plant Material to Understand Agricultural Spread in Prehistory. Plant Genet. Resour..

[47] Bhullar N.K., Street K., Mackay M., Yahiaoui N., Keller B. (2009). Unlocking Wheat Genetic Resources for the Molecular Identification of Previously Undescribed Functional Alleles at the Pm3 Resistance Locus. Proc. Natl. Acad. Sci. USA.

[48] Stephen Baenziger P., Depauw R.M. (2009). Wheat Breeding: Procedures and Strategies. Wheat Science and Trade.

[49] Clark S. (2014). Plant Guide for White Lupine (Lupinus albus L.).

[50] Annicchiarico P., de Buck A.J., Vlachostergios D.N., Heupink D., Koskosidis A., Nazzicari N., Crosta M. (2023). White Lupin Adaptation to Moderately Calcareous Soils: Phenotypic Variation and Genome-Enabled Prediction. Plants.

[51] Kezeya Sepngang B., Muel F., Smadja T., Stauss W. (2020). Report on Legume Markets in the EU Deliverable D3.1 of the EU-Project LegValue.

[52] Pilorgé E., Muel F. (2016). What Vegetable Oils and Proteins for 2030? Would the Protein Fraction Be the Future of Oil and Protein Crops?. OCL.

[53] Watson C.A., Reckling M., Preissel S., Bachinger J., Bergkvist G., Kuhlman T., Lindström K., Nemecek T., Topp C.F.E., Vanhatalo A. (2017). Grain Legume Production and Use in European Agricultural Systems. Adv. Agron..

[54] European Commission (2018). Market Developments and Policy Evaluation Aspects of the Plant Protein Sector in the EU.

[55] Legume Generation Project. https://www.legumegeneration.eu/.

[56] Belis Project. http://www.belisproject.eu/.

[57] Eurolegume Project. https://eurolegume.utad.pt/.

[58] ABSTRESS Project. https://eu-cap-network.ec.europa.eu/projects/abstress-improving-resistance-legume-crops-combined-abiotic-and-biotic-stress_en.

[59] Projects—The International Legume Society. https://www.legumesociety.org/post_full_width/projects-all/.

[60] LupinChallenge Project. https://cordis.europa.eu/project/id/301550.

[61] Legumes. https://legumesproject.eu/.

[62] ECPGR: Legumes. https://www.ecpgr.org/eva/eva-networks/legumes.

[63] LEGVALUE—Legumehub.Eu. https://www.legumehub.eu/legvalue/.

[64] Legumes Translated. https://www.legumestranslated.eu/.

[65] Sustainable Agriculture Through Legume-Cereal Intercropping. https://www.leguminose.eu/.

[66] Legunet. https://www.legunet.de/.

[67] Bresov Shaping the Future of Organic Breeding & Farming. https://bresov.eu/.

[68] Bellucci E., Benazzo A., Xu C., Bitocchi E., Rodriguez M., Alseekh S., Di Vittori V., Gioia T., Neumann K., Cortinovis G. (2023). Selection and Adaptive Introgression Guided the Complex Evolutionary History of the European Common Bean. Nat. Commun..

[69] ERA-CAPS BEAN ADAPT-Evolution in a Changing Environment: The Genetic Architecture of Adaptation Outside Centers of Domestication of *Phaseolus vulgaris* and P. Coccineus..

[70] Cortinovis G., Oppermann M., Neumann K., Graner A., Gioia T., Marsella M., Alseekh S., Fernie A.R., Papa R., Bellucci E. (2021). Towards the Development, Maintenance, and Standardized Phenotypic Characterization of Single-Seed-Descent Genetic Resources for Common Bean. Curr. Protoc..

[71] Bulut M., Wendenburg R., Bitocchi E., Bellucci E., Kroc M., Gioia T., Susek K., Papa R., Fernie A.R., Alseekh S. (2023). A Comprehensive Metabolomics and Lipidomics Atlas for the Legumes Common Bean, Chickpea, Lentil and Lupin. Plant J..

[72] Kroc M., Tomaszewska M., Czepiel K., Bitocchi E., Oppermann M., Neumann K., Guasch L., Bellucci E., Alseekh S., Graner A. (2021). Towards Development, Maintenance, and Standardized Phenotypic Characterization of Single-Seed-Descent Genetic Resources for Lupins. Curr. Protoc..

[73] Rocchetti L., Gioia T., Logozzo G., Brezeanu C., Pereira L.G., De la Rosa L., Marzario S., Pieri A., Fernie A.R., Alseekh S. (2022). Towards the Development, Maintenance and Standardized Phenotypic Characterization of Single-Seed-Descent Genetic Resources for Chickpea. Curr. Protoc..

[74] Guerra-García A., Gioia T., von Wettberg E., Logozzo G., Papa R., Bitocchi E., Bett K.E. (2021). Intelligent Characterization of Lentil Genetic Resources: Evolutionary History, Genetic Diversity of Germplasm, and the Need for Well-Represented Collections. Curr. Protoc..

[75] The International Legume Society—Official Website of ILS. https://www.legumesociety.org/.

[76] Consuela Lorela Cristiana D., Man S., Chiş S., Pop A., Muste S., Păucean A. (2022). Lupin—A Review of the Chemical Composition, Health Benefits and Its Uses in Bakery and Pastry Products. Hop Med. Plants.

[77] Gladstones J.S., Atkins C.A., Hamblin J. (1998). Distribution, Origin, Taxonomy, History and Importance. Lupins as Crop Plant.

[78] Kurlovich B.S. (2002). Lupins: Geography, Classification, Genetic Resources and Breeding.

[79] Drummond C.S., Eastwood R.J., Miotto S.T.S., Hughes C.E. (2012). Multiple Continental Radiations and Correlates of Diversification in *Lupinus* (Leguminosae): Testing for Key Innovation with Incomplete Taxon Sampling. Syst. Biol..

[80] Susek K., Bielski W., Czyż K.B., Hasterok R., Jackson S.A., Wolko B., Naganowska B. (2019). Impact of Chromosomal Rearrangements on the Interpretation of Lupin Karyotype Evolution. Genes.

[81] Taylor J.L., De Angelis G., Nelson M.N. (2020). How Have Narrow-Leafed Lupin Genomic Resources Enhanced Our Understanding of Lupin Domestication?. The Lupin Genome.

[82] Atchison G.W., Nevado B., Eastwood R.J., Contreras-Ortiz N., Reynel C., Madriñán S., Filatov D.A., Hughes C.E. (2016). Lost Crops of the Incas: Origins of Domestication of the Andean Pulse Crop Tarwi, *Lupinus* Mutabilis. Am. J. Bot..

[83] Garg G., Kamphuis L.G., Bayer P.E., Kaur P., Dudchenko O., Taylor C.M., Frick K.M., Foley R.C., Gao L.L., Aiden E.L. (2022). A Pan-genome and Chromosome-length Reference Genome of Narrow-leafed Lupin (*Lupinus angustifolius*) Reveals Genomic Diversity and Insights into Key Industry and Biological Traits. Plant J..

[84] Iqbal M.M., Huynh M., Udall J.A., Kilian A., Adhikari K.N., Berger J.D., Erskine W., Nelson M.N. (2019). The First Genetic Map for Yellow Lupin Enables Genetic Dissection of Adaptation Traits in an Orphan Grain Legume Crop. BMC Genet..

[85] Kohajdová Z., Karovičová J., Schmidt Š. (2011). Lupin Composition and Possible Use in Bakery—A Review. Czech J. Food Sci..

[86] Sharasia P.L., Garg M.R., Bhanderi B.M. (2018). Pulses and Their By-Products as Animal Feed.

[87] Adamidou S., Nengas I., Grigorakis K., Nikolopoulou D., Jauncey K. (2011). Chemical Composition and Antinutritional Factors of Field Peas (*Pisum sativum*), Chickpeas (*Cicer arietinum*), and Faba Beans (*Vicia faba*) as Affected by Extrusion Preconditioning and Drying Temperatures. Cereal Chem..

[88] Badjona A., Bradshaw R., Millman C., Howarth M., Dubey B. (2024). Faba Beans Protein as an Unconventional Protein Source for the Food Industry: Processing Influence on Nutritional, Techno-Functionality, and Bioactivity. Food Rev. Int..

[89] Gulisano A., Alves S., Martins J.N., Trindade L.M. (2019). Genetics and Breeding of *Lupinus* Mutabilis: An Emerging Protein Crop. Front. Plant Sci..

[90] Carvajal-Larenas F.E., Linnemann A.R., Nout M.J.R., Koziol M., van Boekel M.A.J.S. (2016). *Lupinus* Mutabilis: Composition, Uses, Toxicology, and Debittering. Crit. Rev. Food Sci. Nutr..

[91] Pereira A., Ramos F., Sanches Silva A. (2022). Lupin (*Lupinus albus* L.) Seeds: Balancing the Good and the Bad and Addressing Future Challenges. Molecules.

[92] Celmeli T., Sari H., Canci H., Sari D., Adak A., Eker T., Toker C. (2018). The Nutritional Content of Common Bean (*Phaseolus vulgaris* L.) Landraces in Comparison to Modern Varieties. Agronomy.

[93] Rybiński W., Święcicki W., Bocianowski J., Börner A., Starzycka-Korbas E., Starzycki M. (2018). Variability of Fat Content and Fatty Acids Profiles in Seeds of a Polish White Lupin (*Lupinus albus* L.) Collection. Genet. Resour. Crop Evol..

[94] Arnoldi A., Boschin G., Zanoni C., Lammi C. (2015). The Health Benefits of Sweet Lupin Seed Flours and Isolated Proteins. J. Funct. Foods.

[95] Boschin G., Arnoldi A. (2011). Legumes Are Valuable Sources of Tocopherols. Food Chem..

[96] Fontanari G.G., Batistuti J.P., Da Cruz R.J., Saldiva P.H.N., Arêas J.A.G. (2012). Cholesterol-Lowering Effect of Whole Lupin (*Lupinus albus*) Seed and Its Protein Isolate. Food Chem..

[97] Wink M., Botschen F., Gosmann C., Schäfer H., Waterman P.G. (2010). Chemotaxonomy Seen from a Phylogenetic Perspective and Evolution of Secondary Metabolism. Biochemistry of Plant Secondary Metabolism.

[98] Frick K.M., Kamphuis L.G., Siddique K.H.M., Singh K.B., Foley R.C. (2017). Quinolizidine Alkaloid Biosynthesis in Lupins and Prospects for Grain Quality Improvement. Front. Plant Sci..

[99] Unkovich M., Baldock J., Forbes M. (2010). Variability in Harvest Index of Grain Crops and Potential Significance for Carbon Accounting: Examples from Australian Agriculture. Adv. Agron..

[100] Xu W., Zhang Q., Yuan W., Xu F., Muhammad Aslam M., Miao R., Li Y., Wang Q., Li X., Zhang X. (2020). The Genome Evolution and Low-Phosphorus Adaptation in White Lupin. Nat. Commun..

[101] Pueyo J.J., Quiñones M.A., Coba de la Peña T., Fedorova E.E., Lucas M.M. (2021). Nitrogen and Phosphorus Interplay in Lupin Root Nodules and Cluster Roots. Front. Plant Sci..

[102] Le Thanh T., Hufnagel B., Soriano A., Divol F., Brottier L., Casset C., Péret B., Doumas P., Marquès L. (2021). Dynamic Development of White Lupin Rootlets Along a Cluster Root. Front. Plant Sci..

[103] Cheng L., Bucciarelli B., Liu J., Zinn K., Miller S., Patton-Vogt J., Allan D., Shen J., Vance C.P. (2011). White Lupin Cluster Root Acclimation to Phosphorus Deficiency and Root Hair Development Involve Unique Glycerophosphodiester Phosphodiesterases. Plant Physiol..

[104] Xia T., Zhu X., Zhan Y., Liu B., Zhou X., Zhang Q., Xu W. (2024). The White Lupin Trehalase Gene LaTRE1 Regulates Cluster Root Formation and Function under Phosphorus Deficiency. Plant Physiol..

[105] Quiñones M.A., Lucas M.M., Pueyo J.J. (2022). Adaptive Mechanisms Make Lupin a Choice Crop for Acidic Soils Affected by Aluminum Toxicity. Front. Plant Sci..

[106] Hufnagel B., Soriano A., Taylor J., Divol F., Kroc M., Sanders H., Yeheyis L., Nelson M., Péret B. (2021). Pangenome of White Lupin Provides Insights into the Diversity of the Species. Plant Biotechnol. J..

[107] Hufnagel B., Marques A., Soriano A., Marquès L., Divol F., Doumas P., Sallet E., Mancinotti D., Carrere S., Marande W. (2020). High-Quality Genome Sequence of White Lupin Provides Insight into Soil Exploration and Seed Quality. Nat. Commun..

[108] Zafeiriou I., Polidoros A.N., Baira E., Kasiotis K.M., Machera K., Mylona P.V. (2021). Mediterranean White Lupin Landraces as a Valuable Genetic Reserve for Breeding. Plants.

[109] Annicchiarico P., Romani M., Pecetti L. (2018). White Lupin (*Lupinus albus*) Variation for Adaptation to Severe Drought Stress. Plant Breed..

[110] Schwertfirm G., Schneider M., Haase F., Riedel C., Lazzaro M., Ruge-Wehling B., Schweizer G. (2024). Genome-Wide Association Study Revealed Significant SNPs for Anthracnose Resistance, Seed Alkaloids and Protein Content in White Lupin. Theor. Appl. Genet..

[111] Lucas M.M., Stoddard F.L., Annicchiarico P., Frías J., Martínez-Villaluenga C., Sussmann D., Duranti M., Seger A., Zander P.M., Pueyo J.J. (2015). The Future of Lupin as a Protein Crop in Europe. Front. Plant Sci..

[112] FAOSTAT https://www.fao.org/faostat/en/#data/QCL/visualize.

[113] Caramona A., Martins A.M., Seixas J., Marto J. (2024). The Use, Reuse and Valorization of Lupin and Its Industry by-Products for Dermocosmetics Applications. Sustain. Chem. Pharm..

[114] DBRM Lupin Market Size, Share, Industry Trends & Forecast 2029. https://www.databridgemarketresearch.com/reports/global-lupin-market.

[115] Wolko B., Clements J.C., Naganowska B., Nelson M.N., Yang H. (2010). Lupinus. Wild Crop Relatives: Genomic and Breeding Resources.

[116] Tanwar U.K., Tomaszewska M., Czepiel K., Neji M., Jamil H., Rocchetti L., Pieri A., Bitocchi E., Bellucci E., Pipan B. (2024). Genetic and Phenotypic Characterization of Global *Lupinus albus* Genetic Resources for the Development of a CORE Collection. bioRxiv.

[117] Schmutz J., Cannon S.B., Schlueter J., Ma J., Mitros T., Nelson W., Hyten D.L., Song Q., Thelen J.J., Cheng J. (2010). Genome Sequence of the Palaeopolyploid Soybean. Nature.

[118] Susek K., Franco E., Tomaszewska M., Kroc M., Jamil H., Tanwar U., Nelson M.N., Papa R., Delledonne M., Jackson S.A. (2024). The Unexplored Diversity of Wild Lupins Provides Rich Genomic Resources and Insights into Lupin Evolution. bioRxiv.

[119] Cortinovis G., Vincenzi L., Anderson R., Marturano G., Marsh J.I., Bayer P.E., Rocchetti L., Frascarelli G., Lanzavecchia G., Pieri A. (2024). Adaptive Gene Loss in the Common Bean Pan-Genome during Range Expansion and Domestication. Nat. Commun..

[120] Raman R., Cowley R.B., Raman H., Luckett D.J. (2014). Analyses Using SSR and DArT Molecular Markers Reveal That Ethiopian Accessions of White Lupin (*Lupinus albus* L.) Represent a Unique Genepool. Open J. Genet..

[121] Kinghorn A.D., Hussain R.A., Robbins E.F., Balandrin M.F., Stirton C.H., Evans S.V. (1988). Alkaloid Distribution in Seeds of Ormosia, Pericopsis and Haplormosia. Phytochemistry.

[122] Wink M., Mohamed G.I.A. (2003). Evolution of Chemical Defense Traits in the Leguminosae: Mapping of Distribution Patterns of Secondary Metabolites on a Molecular Phylogeny Inferred from Nucleotide Sequences of the RbcL Gene. Biochem. Syst. Ecol..

[123] Mancinotti D., Czepiel K., Taylor J.L., Galehshahi H.G., Møller L.A., Jensen M.K., Motawia M.S., Hufnagel B., Soriano A., Yeheyis L. (2023). The Causal Mutation Leading to Sweetness in Modern White Lupin Cultivars. Sci. Adv..

[124] Kroc M., Rybiński W., Wilczura P., Kamel K., Kaczmarek Z., Barzyk P., Święcicki W. (2017). Quantitative and Qualitative Analysis of Alkaloids Composition in the Seeds of a White Lupin (*Lupinus albus* L.) Collection. Genet. Resour. Crop Evol..

[125] Valente I.M., Sousa C., Almeida M., Miranda C., Pinheiro V., Garcia-Santos S., Ferreira L.M.M., Guedes C.M., Maia M.R.G., Cabrita A.R.J. (2023). Insights from the Yield, Protein Production, and Detailed Alkaloid Composition of White (*Lupinus albus*), Narrow-Leafed (*Lupinus angustifolius*), and Yellow (*Lupinus luteus*) Lupin Cultivars in the Mediterranean Region. Front. Plant Sci..

[126] Gresta F., Oteri M., Scordia D., Costale A., Armone R., Meineri G., Chiofalo B. (2023). White Lupin (*Lupinus albus* L.), an Alternative Legume for Animal Feeding in the Mediterranean Area. Agriculture.

[127] Schrenk D., Bodin L., Chipman J.K., del Mazo J., Grasl-Kraupp B., Hogstrand C., Hoogenboom L., Leblanc J.C., Nebbia C.S., Nielsen E. (2019). Scientific Opinion on the Risks for Animal and Human Health Related to the Presence of Quinolizidine Alkaloids in Feed and Food, in Particular in Lupins and Lupin-Derived Products. EFSA J..

[128] Mancinotti D., Frick K.M., Geu-Flores F. (2022). Biosynthesis of Quinolizidine Alkaloids in Lupins: Mechanistic Considerations and Prospects for Pathway Elucidation. Nat. Prod. Rep..

[129] Harrison J.E.M., Williams W. (1982). Genetical Control of Alkaloids in *Lupinus albus*. Euphytica.

[130] Osorio C.E., Till B.J. (2022). A Bitter-Sweet Story: Unraveling the Genes Involved in Quinolizidine Alkaloid Synthesis in *Lupinus albus*. Front. Plant Sci..

[131] Lin R., Renshaw D., Luckett D., Clements J., Yan G., Adhikari K., Buirchell B., Sweetingham M., Yang H. (2009). Development of a Sequence-Specific PCR Marker Linked to the Gene “Pauper” Conferring Low-Alkaloids in White Lupin (*Lupinus albus* L.) for Marker Assisted Selection. Mol. Breed..

[132] Rychel S., Książkiewicz M. (2019). Development of Gene-Based Molecular Markers Tagging Low Alkaloid Pauper Locus in White Lupin (*Lupinus albus* L.). J. Appl. Genet..

[133] Rodés-Bachs C., Van der Fels-Klerx H.J. (2023). Impact of Environmental Factors on the Presence of Quinolizidine Alkaloids in Lupins: A Review. Food Addit. Contam. Part A.

[134] Jansen G., Jürgens H.-U., Schliephake E., Ordon F. (2012). Effect of the Soil PH on the Alkaloid Content of *Lupinus angustifolius*. Int. J. Agron..

[135] Valente I.M., Monteiro A., Sousa C., Miranda C., Maia M.R.G., Castro C., Cabrita A.R.J., Trindade H., Fonseca A.J.M. (2024). Agronomic, Nutritional Traits, and Alkaloids of *Lupinus albus*, *Lupinus angustifolius* and *Lupinus luteus* Genotypes: Effect of Sowing Dates and Locations. ACS Agric. Sci. Technol..

[136] Annicchiarico P., Harzic N., Huyghe C., Carroni A.M. (2011). Ecological Classification of White Lupin Landrace Genetic Resources. Euphytica.

[137] Brand J.D., Tang C., Rathjen A.J. (2002). Screening Rough-Seeded Lupins (*Lupinus pilosus* Murr. and *Lupinus atlanticus* Glads.) for Tolerance to Calcareous Soils. Plant Soil.

[138] Tang C., Robson A.D., Longnecker N.E., Buirchell B.J. (1995). The Growth of *Lupinus* Species on Alkaline Soils. Aust. J. Agric. Res..

[139] Tang C., Buirchell B.J., Longnecker N.E., Robson A.D. (1993). Variation in the Growth of Lupin Species and Genotypes on Alkaline Soil. Plant Soil.

[140] Annicchiarico P., Harzic N., Carroni A.M. (2010). Adaptation, Diversity, and Exploitation of Global White Lupin (*Lupinus albus* L.) Landrace Genetic Resources. Field Crops Res..

[141] Arief O.M., Pang J., Shaltout K.H., Lambers H. (2020). Performance of Two *Lupinus albus* L. Cultivars in Response to Three Soil PH Levels. Exp. Agric..

[142] Christiansen J.L., Raza S., Jørnsgård B., Mahmoud S.A., Ortiz R. (2000). Potential of Landrace Germplasm for Genetic Enhancement of White Lupin in Egypt. Genet. Resour. Crop Evol..

[143] Raza S., Abdel-Wahab A., Jørnsgård B., Christiansen J.L. Calcium Tolerance and Ion Uptake of Egyptian Lupin Landraces on Calcareous Soils. https://www.bioline.org.br/request?cs01021.

[144] Kerley S.J., Norgaard C., Leach J.E., Christiansen J.L., Huyghe C., Römer P. (2002). The Development of Potential Screens Based on Shoot Calcium and Iron Concentrations for the Evaluation of Tolerance in Egyptian Genotypes of White Lupin (*Lupinus albus* L.) to Limed Soils. Ann. Bot..

[145] Liu A., Tang C. (1999). Comparative Performance of *Lupinus albus* Genotypes in Response to Soil Alkalinity. Aust. J. Agric. Res..

[146] Koseoglou E. Selection of White Lupine Genotypes for Yield and Tolerance to Alkaline Soils. Proceedings of the General EUCARPIA Congress “Plant Breeding for Future Generations”.

[147] Annicchiarico P., Alami I.T. (2012). Enhancing White Lupin (*Lupinus albus* L.) Adaptation to Calcareous Soils through Selection of Lime-Tolerant Plant Germplasm and Bradyrhizobium Strains. Plant Soil.

[148] Dinkelaker B., Römheld V., Marschner H. (1989). Citric Acid Excretion and Precipitation of Calcium Citrate in the Rhizosphere of White Lupin (*Lupinus albus* L.). Plant Cell Environ..

[149] Tang C., Thomson B.D. (1996). Effects of Solution Ph and Bicarbonate on the Growth and Nodulation of a Range of Grain Legume Species. Plant Soil.

[150] Kerley S.J. (2000). The Effect of Soil Liming on Shoot Development, Root Growth, and Cluster Root Activity of White Lupin. Biol. Fertil. Soils.

[151] Tang C., Zheng S.J., Qiao Y.F., Wang G.H., Han X.Z. (2006). Interactions between High PH and Iron Supply on Nodulation and Iron Nutrition of *Lupinus albus* L. Genotypes Differing in Sensitivity to Iron Deficiency. Plant Soil.

[152] Kerley S.J., Huyghe C. (2001). Comparison of Acid and Alkaline Soil and Liquid Culture Growth Systems for Studies of Shoot and Root Characteristics of White Lupin (*Lupinus albus* L.) Genotypes. Plant Soil.

[153] Vasconcelos M., Eckert H., Arahana V., Graef G., Grusak M.A., Clemente T. (2006). Molecular and Phenotypic Characterization of Transgenic Soybean Expressing the Arabidopsis Ferric Chelate Reductase Gene, FRO2. Planta.

[154] Tian Q., Zhang X., Yang A., Wang T., Zhang W.H. (2016). CIPK23 Is Involved in Iron Acquisition of Arabidopsis by Affecting Ferric Chelate Reductase Activity. Plant Sci..

[155] Pestana M., David M., de Varennes A., Abadía J., Faria E.A. (2001). Responses of “Newhall” Orange Trees to Iron Deficiency in Hydroponics: Effects on Leaf Chlorophyll, Photosynthetic Efficiency, and Root Ferric Chelate Reductase Activity. J. Plant Nutr..

[156] Zhao Y., Liu S., Li F., Sun M., Liang Z., Sun Z., Yu F., Li H. (2023). The Low Ferric Chelate Reductase Activity and High Apoplastic PH in Leaves Cause Iron Deficiency Chlorosis in ‘Huangguan’ Pears Grafted onto Quince A Grown in Calcareous Soil. Sci. Hortic..

[157] Ishimaru Y., Kim S., Tsukamoto T., Oki H., Kobayashi T., Watanabe S., Matsuhashi S., Takahashi M., Nakanishi H., Mori S. (2007). Mutational Reconstructed Ferric Chelate Reductase Confers Enhanced Tolerance in Rice to Iron Deficiency in Calcareous Soil. Proc. Natl. Acad. Sci. USA.

[158] Mori S. (1999). Iron Acquisition by Plants. Curr. Opin. Plant Biol..

[159] Ceccarelli S. (2011). Landraces: Importance and Use in Breeding and Environmentally Friendly Agronomic Systems. Agrobiodiversity Conservation: Securing the Diversity of Crop Wild Relatives and Landraces.

[160] Dwivedi S.L., Ceccarelli S., Blair M.W., Upadhyaya H.D., Are A.K., Ortiz R. (2016). Landrace Germplasm for Improving Yield and Abiotic Stress Adaptation. Agrobiodiversity Conservation: Securing the Diversity of Crop Wild Relatives and Landraces.

[161] Ceccarelli S. (1997). Adaptation to Low/High Input Cultivation. Euphytica.

[162] Ceccarelli S. (1996). Positive Interpretation of Genotype by Environment Interaction in Relation to Sustainability and Biodiversity. Plant Adaptation and Crop Improvement.

[163] Sharma S., Upadhyaya H.D., Varshney R.K., Gowda C.L.L. (2013). Pre-Breeding for Diversification of Primary Gene Pool and Genetic Enhancement of Grain Legumes. Front. Plant Sci..

[164] Pratap A., Das A., Kumar S., Gupta S. (2021). Current Perspectives on Introgression Breeding in Food Legumes. Front. Plant Sci..

[165] Abraham E.M., Ganopoulos I., Madesis P., Mavromatis A., Mylona P., Nianiou-Obeidat I., Parissi Z., Polidoros A., Tani E., Vlachostergios D. (2019). The Use of Lupin as a Source of Protein in Animal Feeding: Genomic Tools and Breeding Approaches. Int. J. Mol. Sci..

[166] Mavromatis A., Nianiou-Obeidat I., Polidoros A., Parissi Z., Tani E., Irakli M., Aliferis K.A., Zafeiriou I., Mylona P.V., Sarri E. (2023). Characterization of Lupin Cultivars Based on Phenotypical, Molecular and Metabolomic Analyses. Agronomy.

[167] Lazaridi E., Kapazoglou A., Gerakari M., Kleftogianni K., Passa K., Sarri E., Papasotiropoulos V., Tani E., Bebeli P.J. (2024). Crop Landraces and Indigenous Varieties: A Valuable Source of Genes for Plant Breeding. Plants.

[168] Marone D., Russo M.A., Mores A., Ficco D.B.M., Laidò G., Mastrangelo A.M., Borrelli G.M. (2021). Importance of Landraces in Cereal Breeding for Stress Tolerance. Plants.

[169] Zhou Y., Zhang Z., Bao Z., Li H., Lyu Y., Zan Y., Wu Y., Cheng L., Fang Y., Wu K. (2022). Graph Pangenome Captures Missing Heritability and Empowers Tomato Breeding. Nature.

[170] Simmonds N.W. (1993). Introgression and incorporation. Strategies for the use of crop genetic resources. Biol. Rev..

[171] Cheng S., Feng C., Wingen L.U., Cheng H., Riche A.B., Jiang M., Leverington-Waite M., Huang Z., Collier S., Orford S. (2024). Harnessing Landrace Diversity Empowers Wheat Breeding. Nature.

[172] Huang K., Jahani M., Gouzy J., Legendre A., Carrere S., Lázaro-Guevara J.M., González Segovia E.G., Todesco M., Mayjonade B., Rodde N. (2023). The Genomics of Linkage Drag in Inbred Lines of Sunflower. Proc. Natl. Acad. Sci. USA.

[173] Smýkal P., Coyne C., Von Wettberg E., Hübner S., Kantar M.B. (2021). Tapping Diversity from the Wild: From Sampling to Implementation. Front. Plant Sci..

[174] Dempewolf H., Baute G., Anderson J., Kilian B., Smith C., Guarino L. (2017). Past and Future Use of Wild Relatives in Crop Breeding. Crop Sci..

[175] Nadeem M.A., Nawaz M.A., Shahid M.Q., Doğan Y., Comertpay G., Yıldız M., Hatipoğlu R., Ahmad F., Alsaleh A., Labhane N. (2018). DNA Molecular Markers in Plant Breeding: Current Status and Recent Advancements in Genomic Selection and Genome Editing. Biotechnol. Biotechnol. Equip..

[176] Hospital F. (2001). Size of Donor Chromosome Segments Around Introgressed Loci and Reduction of Linkage Drag in Marker-Assisted Backcross Programs. Genetics.

[177] Zhang F., Shi Y., Ali J., Xu J., Li Z. (2021). Breeding by Selective Introgression: Theory, Practices, and Lessons Learned from Rice. Crop J..

[178] Gupta S., Buirchell B.J., Cowling W.A. (1996). Interspecific Reproductive Barriers and Genomic Similarity among the Rough-Seeded *Lupinus* Species. Plant Breed..

